# Book Reviews

**Published:** 1891-05

**Authors:** 


### Book Reviews.

---

Essentials of Practice of Medicine. By Henry Morris M. D., late Demonstrator Jefferson Medical College, Philadelphia, etc. Philadelphia. W. B. Saunders.

The "Essentials" and "Compends" seem to have come to stay, and doubtless accomplish a certain purpose. The present one is designed especially for the advanced student of medicine and for the young practitioner, and for its size certainly seems to cover the ground remarkably well. Students, we believe, will find the book useful. Necessarily there are important omissions.

The appendix on the Examination of Urine by Dr. Lawrence Wolff is a very fair presentation of the subject in a concise and convenient shape.

---

Bacteriological Technology. By C. J. Salomonsen. Tranlated from the second Danish edition, by William Trelease. Pages 162. William Wood & Co., New York.

In presenting this book to the profession the author had two objects in view (preface): The publication of an outline adapted to bacteriological courses for physicians, and a guide for those who are obliged to take up the subject at home without the assistance of an instructor. This is therefore no elaborate treatise on bacteriology. However, the methods of bacteriological technique, such as sterilization, culture media, inoculation of animals, staining of bacteria, etc., are described with sufficient detail to make the work a very valuable manual. For sometime there has been a demand for a book of this sort for English readers, and we believe that this want is now supplied.

The Year-Book of Treatment for 1891. Lea Brothers & Co. Philadelphia. Pages 480. Price $1.50.

The favorable reception which these Year-Books have received from the profession in the past has encouraged the publishers in the current issue to add considerably to the size of previous ones. The original purpose, namely, to supply a concise epitome of the chief articles of the preceding year, with a short criticism of the more important subjects, has not been altered. The list of contributors is sufficient guarantee. These represent every department of medical art. Diseases of the Heart and Circulation, by Mitchell Bruce; Diseases of the Nervous System, by James Ross; General Surgery, by Stanley Boyd; Diseases of Women, by Berry Hart; Diseases of the Genito-Urinary System, by Reginald Harrison; and so on with other names of equal merit.

---

Materia Medica, Pharmacy and Therapeutics. By Samuel O. L. Potter, M. D., Professor of the Theory and Practice of Medicine in the Cooper Medical College of San Francisco, etc. Second edition. Philadelphia. P. Blankiston, Son & Co.

Perhaps the text-books in no department of medical science are more unsatisfactory than those of Materia Medica. In spite of the classical works of Wood and Ringer and Brunton, it is not too much to say that what the student and practitioner most need has not yet been written. And it was this fact which led Dr. Potter, about five years ago, to prepare the first edition of the above work for the advanced student and junior practitioner. The book is divided into three principal parts, Materia Medica proper, Special Therapeutics of Disease, and Official and Extemporaneous Pharmacy. The author adopts the alphabetical arrangement of drugs, which is not so good as the physiological method of Wood, but better than the botanical arrangement of Brunton. The department of Special Therapeutics is complete and valuable.

We do not hesitate to give our cordial recommendation to this book, and we may congratulate Dr. Potter on the warm reception which has been recorded his work by the profession. We believe the general practitioner will find this to supply his needs more nearly than any other book on the subject with which we are acquainted.

---

ESSENTIALS OF MINOR SURGERY, BANDAGING, AND VENEREAL DISEASES. By EDWARD MARTIN, M. D., Instructor in Operative Surgery, University of Pennsylvania, etc. Pp. 166. Illustrated. W. B. Saunders, Philadelphia.

The subjects usually embraced in "minor surgery" are discussed in this little volume as fully as the allotted space will permit. The modest little book presents in a neat and accessible shape a great deal of useful advice and suggestion not found in large works. Forty-six pages are devoted to chancroid, gonorrhœa and syphilis. In the diagnosis of gonorrhœa the author seems to us a little radical. This, he says, cannot be positively made in the absence of the gonococcus. In the discussion of syphilis we see nothing new.

---

ESSENTIALS OF DISEASES OF CHILDREN. By W. M. POWELL, M. D., Physician to the Clinic for Diseases of Children in the University of Pennsylvania, etc. Pp. 222. W. B. Saunders, Philadelphia.

This little book is a fair presentation of this great subject, and is doubtless all that the author claims for it. The introduction consists of remarks on examination in general, fæcal evacuations, vomiting, the pulse and temperature.

Necessarily diseases of the alimentary canal and acute infectious diseases receive considerable attention. The different diseases are handled with special reference to symptoms, diagnosis and treatment. As far as it goes this "essential" fulfills its purpose.

